# First clinical-epidemiological profile of zoonotic sporotrichosis in a state capital of Northeast Brazil: evidence of an emerging feline-driven urban epidemic

**DOI:** 10.1590/S1678-9946202668069

**Published:** 2026-10-19

**Authors:** Thiago Dutra Mendonça, Rayssa de Sousa Silva, Sirlei Garcia Marques, Gustavo Silva Santos de Sousa, Bianca de Fátima Assunção Sodré, Ayrton Rocha Lima, Guilherme Alencar de Medeiros, Daniel Wagner de Castro Lima Santos, Conceição de Maria Pedrozo e Silva de Azevedo

**Affiliations:** 1 Universidade Federal do Maranhão, Programa de Pós-Graduação em Ciências da Saúde, São Luís, Maranhão, Brazil; 2 Universidade Federal do Maranhão, Hospital Universitário Presidente Dutra, São Luís, Maranhão, Brazil; 3 Laboratório Cedro, São Luís, Maranhão, Brazil; 4 Universidade Federal do Maranhão, Faculdade de Medicina, São Luís, Maranhão, Brazil; 5 Rede D’Or, Instituto D’Or de Pesquisa e Ensino, São Luís, Maranhão, Brazil

**Keywords:** Sporotrichosis, Zoonoses, Cats, Sporothrix, Epidemiology, Public health.

## Abstract

Zoonotic sporotrichosis has expanded in Brazil as an urban, cat-associated mycosis, but its human clinical-epidemiological profile remains poorly characterized in Maranhao State, Brazil. This study describes a hospital-based cohort from Greater Sao Luis and defines a regional profile not previously established in the state. We conducted an observational study combining retrospective and prospective data from 2022 to 2025 at a reference hospital for infectious diseases in Sao Luis municipality, Maranhao State, Brazil. Confirmed cases were defined by culture and/or compatible histopathological findings supported by clinical-epidemiological criteria. Demographic, exposure, clinical, laboratory, therapeutic, and outcome variables were analyzed using descriptive statistics and appropriate statistical tests. A total of 189 confirmed cases were analyzed. Cases increased sharply after 2022 and peaked in 2024, with 82 cases. Most patients lived in Sao Luis municipality (84.7%), were female (67.2%), and were aged 30-59 years (47.1%). Feline-associated exposure predominated: 70.9% reported having household cats and 81.5% reported scratches or contact with feline lesions or secretions. The lymphocutaneous form was the main presentation (61.9%), followed by fixed cutaneous disease (29.1%); upper limbs were the most frequently affected sites (73.0%). Culture was positive for *Sporothrix* spp. in 62.2% of tested samples, and suppurative granulomas were frequent findings on histopathological examination. Itraconazole was the first-line therapy in 92.0% of treated patients. Greater Sao Luis shows the profile of an emerging urban epidemic of zoonotic sporotrichosis of feline origin. These findings provide local evidence to guide surveillance, early diagnosis, integration between human and veterinary health services, and One Health-based control strategies in Maranhao State.

## INTRODUCTION

Sporotrichosis is an implantation mycosis caused by thermodimorphic fungi of the genus *Sporothrix*. Although it was traditionally associated with traumatic inoculation from soil, plant debris, and occupational exposure, its epidemiology has changed substantially in Brazil with the rise of cat-transmitted disease^1^ ^),(^ ^2^. In this setting, infected felines act as efficient amplifiers because their lesions may contain high fungal burden, and transmission can occur through scratches, bites, or contact with exudates^2^ ^),(^ ^3^. Additional transmission routes, including sapronotic exposure, contaminated fomites, and respiratory transmission from infected cats, have also been reported and should be considered in surveillance and prevention strategies^4^ ^),(^ ^5^.

Brazil is currently the most relevant global setting for zoonotic sporotrichosis, particularly because feline-associated transmission in the country has been strongly linked to *Sporothrix brasiliensis*, a highly virulent species that has driven major urban outbreaks^1^ ^),(^ ^2^. The first large urban epidemic was described in Rio de Janeiro city, where sustained transmission among cats and humans transformed sporotrichosis from an occasional implantation mycosis into an urban public health issue^3^. Subsequent reports have documented geographic spread to other Brazilian regions, including areas where systematic human data remain limited^4^ ^),(^ ^6^ ^-^ ^8^.

Sporotrichosis has been reported with increasing frequency in the northeastern region of Brazil, but the human burden and clinical profile of the disease remain unevenly documented^7^. In Maranhao State, recent veterinary and laboratory evidence suggests circulation of *Sporothrix* spp. in the metropolitan area of Sao Luis municipality; however, human data sufficient to define the affected population, main exposures, clinical presentations, diagnostic patterns, and therapeutic response are still scarce. This gap limits surveillance planning, clinical suspicion, and the implementation of integrated human-animal-environment control measures^4^ ^),(^ ^9^ ^),(^ ^10^.

Therefore, this study aims to describe the clinical-epidemiological profile, diagnostic findings, and therapeutic characteristics of confirmed cases of zoonotic sporotrichosis treated at a reference hospital in Sao Luis municipality, Maranhao State, Brazil, from 2022 to 2025. By defining a local profile in a previously undercharacterized region, the study provides evidence for public health action and One Health strategies in the state.

### Ethics

The study was approved by the Research Ethics Committee of the Universidade Federal do Maranhao under Certificate of Presentation for Ethical Appraisal Nº 84202724.9.0000.5087. All procedures followed Brazilian ethical standards for research involving human beings. For retrospective data, informed consent was waived because data were collected from medical records. For prospective data, written informed consent was obtained from participants or their legal guardians. Written informed consent for publication of the facial image in Figure 2 was obtained from the patient. The eyes were obscured for privacy.

## MATERIALS AND METHODS

### Study design and setting

This was an observational study combining retrospective data from January 2022 to December 2023 and prospective data from January 2024 to December 2025. The study was conducted at a reference hospital for infectious and parasitic diseases in Sao Luis municipality, Maranhao State, Brazil, which receives patients from the capital and surrounding municipalities.

### Study population and case definition

The study population comprised patients with confirmed sporotrichosis treated during the study period. Case eligibility was established based on compatible clinical-epidemiological and laboratory criteria, in line with Brazilian surveillance guidance for sporotrichosis^11^. Eligible cases met at least one of the following criteria: positive culture for *Sporothrix* spp.; histopathological findings compatible with sporotrichosis, including suppurative granulomatous inflammation, with or without asteroid bodies or yeast-like structures; or a compatible clinical-epidemiological presentation supported by laboratory or histopathological evidence. Compatible clinical presentations included single or multiple papular, nodular, nodular-ulcerative, cutaneous, or mucosal lesions, with or without lymphatic spread, associated with previous contact with cats, traumatic inoculation, contact with organic material, or other plausible exposure routes. Cases with inconclusive diagnosis or insufficient data for analysis were excluded.

### Data collection

 Data were obtained from medical records and hospital notification forms. Variables included sex, age, municipality of residence, year of diagnosis, contact with cats, scratches or contact with feline lesions or secretions, gardening or soil exposure, clinical form, lesion site, mycological culture, histopathological findings, antifungal treatment, treatment duration, and clinical outcome when available.

### Laboratory diagnosis

Mycological investigation was performed at the Central Public Health Laboratory of Maranhao State and at Cedro Laboratory. Clinical samples, including pus, lesion exudate, and biopsy fragments, were cultured on Sabouraud dextrose agar supplemented with chloramphenicol and cycloheximide (Mycosel-type selective medium) and incubated at 25 °C and 37 °C. Detailed information on the commercial manufacturers was not consistently available for all retrospective samples. Presumptive identification followed routine laboratory criteria based on colony growth and micromorphological features compatible with *Sporothrix* spp*.*; however, detailed macro- and micromorphological descriptors and systematic documentation of yeast conversion at 37 °C were not available for all isolates. Histopathological examination was performed on skin biopsy fragments fixed in 10% formalin and embedded in paraffin. Sections 3-5 μm thick were stained with hematoxylin and eosin for morphological analysis; when indicated, Grocott methenamine silver and periodic acid-Schiff stains were used to detect fungal structures.

### Statistical analysis

Categorical variables were summarized as absolute and relative frequencies. Continuous variables were described as means and standard deviations or medians, according to data distribution. Associations between categorical variables were evaluated using statistical tests. Statistical significance was defined as p<0.05. Analyses were performed using SPSS (version 26.0, IBM Corp., Armonk, NY, USA).

## RESULTS

A total of 189 confirmed cases of zoonotic sporotrichosis were analyzed. The annual distribution showed a marked increase during the study period, with 2024 accounting for the highest number (82 cases), followed by 2023 and 2025 (Figure 1).

**Figure 1:**
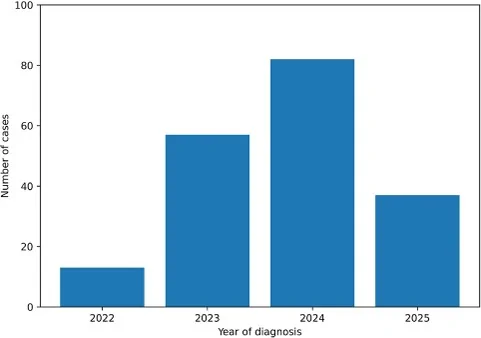
Temporal distribution of confirmed sporotrichosis cases in the hospital-base cohort, Maranhao State, Brazil, 2022-2025.

Most patients lived in Sao Luis municipality, were female, and were adults aged 30-59 years. The mean age was 43.7 years (standard deviation, 17 years). The demographic distribution is summarized in Table 1.

**Table 1:** Demographic profile and annual distribution of confirmed sporotrichosis cases in Greater Sao Luis, Maranhao State, Brazil, 2022-2025.

Characteristic	n	%
Year of diagnosis		
2022	13	6.9
2023	57	30.2
2024	82	43.4
2025	37	19.6
**Sex**		
Female	127	67.2
Male	62	32.8
**Age group, years**		
0-19	28	14.8
20-29	30	15.9
30-59	89	47.1
≥60	42	22.2
**Mean age, years (SD)**	43.7 (17.0)	
**Municipality of residence**		
Sao Luis	160	84.7
Other municipalities in Maranhao State	29	15.3

SD = standard deviation; percentages estimated based on 189 confirmed cases.

Feline-associated exposure was the dominant epidemiological pattern. Household contact with cats was reported by 134 of 189 patients with available data (70.9%), and scratches or contact with feline lesions or secretions were reported by 154 of 189 patients (81.5%). These exposure variables were not mutually exclusive, and household cats were frequently described as sick in records that documented direct contact with feline lesions or secretions. Gardening or soil exposure was uncommon, reported by only seven patients (3.7%).

The lymphocutaneous form was the most frequent clinical presentation, followed by fixed cutaneous disease. Disseminated forms and other presentations, including mucosal and osteoarticular involvement, were less common. Ten disseminated cases were recorded in the dataset; among them, one involved the retro-orbital region, with optic chiasm compression and permanent visual sequelae. Representative clinical presentations are shown in Figure 2. Clinical characteristics and exposure variables are presented in Table 2.

**Figure 2:**
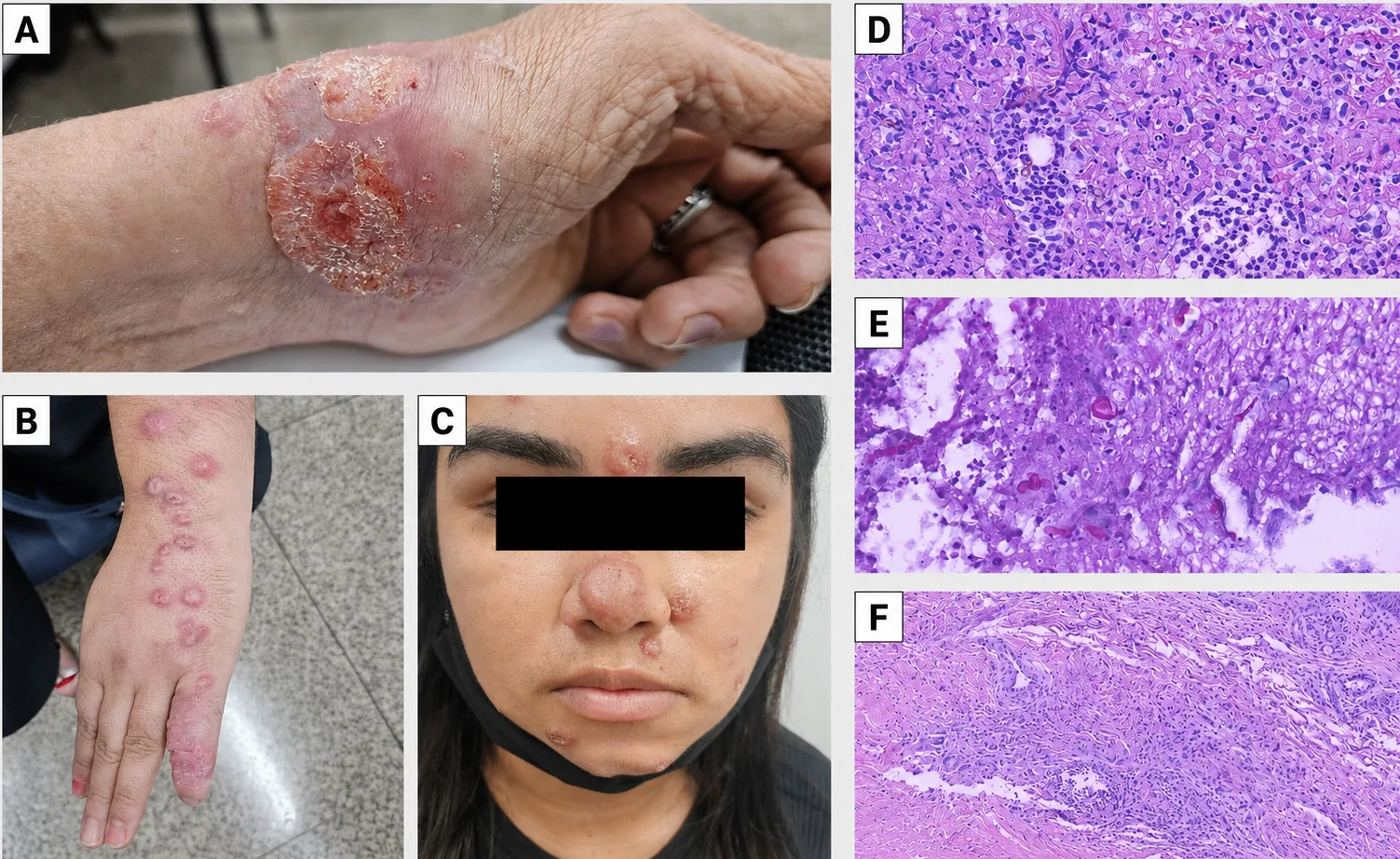
Clinical and histopathological spectrum of zoonotic sporotrichosis: (A) fixed cutaneous sporotrichosis presenting as an ulcerated and crusted plaque on the dorsal aspect of the hand and wrist; (B) lymphocutaneous sporotrichosis characterized by multiple nodular lesions distributed along the lymphatic drainage pathway of the upper limb; (C) disseminated cutaneous involvement with multiple facial lesions affecting the nasal, perioral, and chin regions; the eyes were anonymized for editorial and ethical protection; (D) histopathological section showing a lymphohistiocytic granulomatous inflammatory infiltrate with neutrophils and foci of necrosis; (E) higher-magnification histopathological section showing neutrophilic inflammation with fungal structures interspersed within the inflammatory infiltrate; (F) histopathological section demonstrating granulomatous inflammation with lymphohistiocytic and neutrophilic components and areas of tissue necrosis. Hematoxylin and eosin staining (D-F).

**Table 2:** Epidemiological exposure and clinical characteristics of confirmed sporotrichosis cases.

Characteristic	n/N	%
Epidemiological exposure		
Household cats	134/189	70.9
Scratches or contact with feline lesions/secretions	154/189	81.5
Gardening or soil contact	7/189	3.7
Clinical form		
Lymphocutaneous	117/189	61.9
Fixed cutaneous	55/189	29.1
Disseminated	10/189	5.3
Other forms (mucosal or osteoarticular)	7/189	3.7
Lesion **location**		
Upper limbs	138/189	73.0
Lower limbs	30/189	15.9
Face/neck	15/189	7.9
Trunk	6/189	3.2

Among 135 patients who underwent mycological culture, 84 (62.2%) were positive for *Sporothrix* spp*.* Histopathology was available for 60 patients, and suppurative granulomas were found in 40 (66.7%). Multinucleated giant cells and acute inflammatory infiltrates were frequent, and fungal structures were observed in selected cases. Itraconazole was used in 92.1% of treated patient, used in 174 of 189 treated patients (92.0%); alternative regimens recorded in a minority of cases included potassium iodide and terbinafine. Most regimens lasted three months. Hospitalization, standardized outcome assessment, and detailed reasons for treatment modification were not consistently documented in the medical records and therefore could not be robustly analyzed. Table 3 shows diagnostic and therapeutic data.

## DISCUSSION

This study defines a clinical-epidemiological profile of zoonotic sporotrichosis in Greater Sao Luis, Maranhao State, Brazil, and supports the interpretation that the disease is expanding as an urban mycosis of feline origin in the region. The increase in cases from 2022 to 2024, together with the predominance of cat-related exposure and localized cutaneous forms, resembles the pattern reported in other Brazilian urban centers affected by feline-transmitted sporotrichosis^2^ ^),(^ ^7^.

The concentration of cases in Sao Luis municipality indicates that the capital may represent an important focus of transmission or diagnosis. This finding may reflect a combination of factors, including population density, close contact between humans and domestic or stray cats, better access to a reference hospital, and greater clinical recognition. Nevertheless, the magnitude of the increase suggests that the phenomenon cannot be explained solely by referral bias and deserves structured surveillance beyond hospital detection.

**Table 3:** Diagnostic findings and therapeutic characteristics of patients with sporotrichosis

Characteristic	n/N	%
Mycological culture		
Positive for *Sporothrix* spp.	84/135	62.2
Negative	51/135	37.8
**Histopathology**		
Suppurative granuloma	40/60	66.7
Other findings	20/60	33.3
**Medication used**		
Itraconazole	174/189	92.0
Potassium iodide	10/189	5.3
Terbinafine	5/189	2.7
**Treatment duration**		
3 months	109/189	57.7
4-6 months	60/189	31.7
>6 months	20/189	10.6

Female predominance contrasts with the historical occupational form of sporotrichosis, classically associated with rural or agricultural exposure. However, it is consistent with the contemporary zoonotic profile observed in Brazil, in which women may have greater exposure as caregivers of sick cats in the household environment^2^ ^),(^ ^3^ ^),(^ ^12^. The predominance among adults aged 30-59 years also suggests involvement of the economically active population, with possible effects on productivity, family routines, and caregiving dynamics.

The epidemiological findings support the role of feline transmission. More than two-thirds of patients reported having household cats, and more than 80% reported scratches or contact with feline lesions or secretions. In contrast, gardening or soil exposure was rare. This contrast highlights the transition from sapronotic or occupational sporotrichosis to a predominantly urban zoonosis in the studied setting^1^ ^),(^ ^2^ ^),(^ ^13^. In Maranhao State, this distinction is important because control cannot rely only on individual protection against soil exposure; it requires early diagnosis and treatment of infected cats, responsible pet ownership, safe handling of sick animals, and coordinated surveillance between human and veterinary health services.

The clinical pattern was also consistent with feline-associated inoculation. The predominance of lymphocutaneous and fixed cutaneous forms, together with frequent upper-limb involvement, suggests direct traumatic inoculation followed by local or lymphatic spread. Disseminated presentations were uncommon but clinically relevant, particularly the case with retro-orbital involvement and permanent visual sequelae. Although most cutaneous sporotrichosis cases are not life-threatening, severe presentations reinforce the need for early recognition, adequate treatment, and referral pathways for complicated disease. Information on comorbidities and immune status was incomplete, which hinders deeper interpretation of disseminated presentations in this cohort^4^ ^),(^ ^14^.

Culture remains central for diagnostic confirmation, but histopathology was useful in this cohort. Suppurative granulomatous inflammation, neutrophilic infiltrates, and occasional fungal structures were compatible with active fungal infection. These findings are particularly relevant in settings where culture or molecular identification is not widely available, although histopathology alone should be interpreted together with clinical and epidemiological evidence. Published sensitivity estimates for histopathology in sporotrichosis are often modest, and our findings should be interpreted in the context of a referral-center cohort and selective submission of biopsies rather than as a direct measure of diagnostic sensitivity^4^ ^),(^ ^5^.

Itraconazole was the main therapeutic agent, consistent with international recommendations for fixed cutaneous and lymphocutaneous sporotrichosis^4^ ^),(^ ^14^. The predominance of three-month treatments may indicate early diagnosis and favorable response in many patients. However, according to recommendations, treatment should ideally continue beyond clinical resolution, and recurrence, adherence, access to medication, hospitalization, and loss to follow-up could not be systematically evaluated in this dataset and should be investigated in future studies.

### Limitations

This study has limitations. Its hospital-based design may overrepresent patients who accessed specialized care and underrepresent mild cases treated elsewhere or not diagnosed in the community. The retrospective component depended on the completeness of medical records and notification forms, resulting in variable denominators in some analyses and incomplete information on hospitalization, immune status, standardized outcomes, and indications for treatment modification. Full georeferenced residential data were also unavailable, limiting a more detailed spatial analysis. Molecular confirmation at the species level was not available for all isolates; therefore, *Sporothrix brasiliensis* is discussed as the most plausible species in the Brazilian zoonotic context and in light of local feline evidence, but not as a confirmed species for all human cases.

Despite these limitations, this study provides local evidence from a poorly characterized area and has immediate public health implications. Greater Sao Luis requires integrated surveillance of human and feline sporotrichosis, active case finding in areas with infected cats, professional training for early diagnosis, veterinary diagnosis and treatment of sick animals, patient education on safe animal handling, and intersectoral One Health strategies to interrupt transmission. These measures should be coupled with systematic notification, public communication, and feline population management policies.

## CONCLUSION

Zoonotic sporotrichosis in Greater Sao Luis has the profile of an emerging urban epidemic of feline origin, marked by an increasing number of cases, predominance among adult women, intense household exposure to cats, frequent scratches or contact with feline lesions, and predominantly lymphocutaneous disease affecting the upper limbs. To the best of our knowledge, this hospital-based cohort provides the first regional clinical-epidemiological characterization of human sporotrichosis in this setting and offers actionable evidence for surveillance and control. The findings support the urgent need for One Health strategies integrating human healthcare, veterinary diagnosis and treatment, feline population management, health education, systematic notification, and coordinated surveillance to contain the expansion of sporotrichosis in Maranhao State and the Brazilian Northeast.

## Data Availability

The authors will not make additional data available due to ethical restrictions.

## References

[1] Rodrigues AM, Della Terra PP, Gremião ID, Pereira SA, Orofino-Costa R, Camargo ZP (2020). The threat of emerging and re-emerging pathogenic Sporothrix species. Mycopathologia.

[2] Gremião ID, Miranda LH, Reis EG, Rodrigues AM, Pereira SA (2017). Zoonotic epidemic of sporotrichosis: cat to human transmission. PLoS Pathog.

[3] Barros MB, Schubach AO, Valle AC, Galhardo MC, Conceição-Silva F, Schubach TM (2004). Cat-transmitted sporotrichosis epidemic in Rio de Janeiro, Brazil: description of a series of cases. Clin Infect Dis.

[4] Rodrigues AM, Gonçalves SS, Carvalho JA, Borba-Santos LP, Rozental S, Camargo ZP (2022). Current progress on epidemiology, diagnosis, and treatment of sporotrichosis and their future trends. J Fungi (Basel).

[5] Orofino-Costa R, Freitas DF, Bernardes-Engemann AR, Rodrigues AM, Talhari C, Ferraz CE (2022). Human sporotrichosis: recommendations from the Brazilian Society of Dermatology for clinical, diagnostic and therapeutic management. An Bras Dermatol.

[6] Gremião ID, Oliveira MM, Miranda LH, Freitas DF, Pereira SA (2020). Geographic expansion of sporotrichosis, Brazil. Emerg Infect Dis.

[7] Silva CE, Valeriano CA, Ferraz CE, Neves RP, Oliveira MM, Silva JC (2021). Epidemiological features and geographical expansion of sporotrichosis in the state of Pernambuco, northeastern Brazil. Future Microbiol.

[8] Cognialli RC, Cáceres DH, Bastos FA, Cavassin FB, Lustosa BP, Vicente VA (2023). Rising incidence of Sporothrix brasiliensis infections, Curitiba, Brazil, 2011-2022. Emerg Infect Dis.

[9] Barros AF, Vitor JS, Esposito YR, Oliveira NS, Silva EM, Souza IM (2025). Phylogenetic analysis of Sporothrix brasiliensis isolated from feline sporotrichosis on São Luís Island, Maranhão, Brazil. Pesq Vet Bras.

[10] Brasil. Ministério da Saúde. Secretaria de Vigilância em Saúde e Ambiente. Departamento de Doenças Transmissíveis. Coordenação-Geral de Vigilância de Zoonoses e Doenças de Transmissão Vetorial Nota Técnica nº 60/2023-CGZV/DEDT/SVSA/MS: a respeito das recomendações sobre a vigilância da esporotricose animal no Brasil.

[11] Brasil. Ministério da Saúde. Secretaria de Vigilância em Saúde e Ambiente. Departamento de Ações Estratégicas de Epidemiologia e Vigilância em Saúde e Ambiente (2024). Guia de vigilância em saúde: volume 3.

[12] Chakrabarti A, Bonifaz A, Gutierrez-Galhardo MC, Mochizuki T, Li S (2015). Global epidemiology of sporotrichosis. Med Mycol.

[13] Etchecopaz A, Toscanini MA, Gisbert A, Mas J, Scarpa M, Iovannitti CA (2021). Sporothrix brasiliensis: a review of an emerging South American fungal pathogen, its related disease, presentation and spread in Argentina. J Fungi (Basel).

[14] Kauffman CA, Bustamante B, Chapman SW, Pappas PG (2007). Clinical practice guidelines for the management of sporotrichosis: 2007 update by the Infectious Diseases Society of America. Clin Infect Dis.

